# Malignant melanoma of the Breast: Case Report

**DOI:** 10.51894/001c.144636

**Published:** 2025-09-30

**Authors:** Raquel Rudy

**Affiliations:** 1 Department of Internal Medicine Henry Ford Warren

## 118

### INTRODUCTION

Malignant melanoma is an aggressive originating from melanocytes, with the potential of metastasizing to any part of the body, it rarely occurs on the breast (1, 2, 4, 6, 11, 12, 15). When malignant melanoma of breast tissue (MIBT) is first detected, it is commonly mistaken for other cancers or benign breast disease. If not caught quickly, it may result in a giant melanoma, which is greater than 10 centimeters in size.

### CASE PRESENTATION

A 55-year-old female with family history of breast cancer presented with an enlarging left breast mass, associated with malodorous purulent drainage. On examination, the mass measured 10cm X 3cm, was fungating, and exhibited purulent drainage. CT scan of chest revealed a left anterior chest wall soft tissue mass with bilobed axillary lymphadenopathy. IV Unasyn and Vancomycin were initiated with topical Flagyl for superimposed bacterial infection. Staging workup including CT abdomen/pelvis, MRI brain, and nuclear medicine whole body bone scan were negative for metastases; however, PET scan showed hypermetabolic left chest wall mass, larger left axillary mass, left axillary lymph node, and right axillary lymph node, suspicious for metastasis. Pathologic biopsy, immunohistochemical stains and next generation sequencing confirmed malignant melanoma. Treatment plan consisted of radiation therapy and immunotherapy to reduce mass size for surgical removal.

### DISCUSSION

Malignant melanoma most commonly affects the skin but rarely breast tissue, occurring in less than 5% (2, 4, 6, 11, 12). There are four categories of MIBT based on origins. The first two categories represent melanomas that originate from the breast tissue, known as primary malignant melanoma of the breast (PMMB), occurring 3-5% of melanomas and accounting for 0.5% of all breast cancers (3, 9). Work-up includes patient history, physical exam, multiple imaging modalities, and biopsy with pathologic morphology, immunohistochemistry and genetic sequencing. The most common positive immunohistochemical markers are S-100 protein, Melan-A, and HMB-45 (3, 6, 9, 15). BRAF V600E can also support the diagnosis and guide treatment (3, 7, 9, 11, 13, 15). This case highlights the importance of rapid and accurate diagnosis to ensure timely patient education, promote adherence to treatment, and prevent delays in care.

